# Are There Any Common Genetic Risk Markers for Rheumatoid Arthritis and Periodontal Diseases? A Case-Control Study

**DOI:** 10.1155/2019/2907062

**Published:** 2019-02-12

**Authors:** Susanne Schulz, Natalie Pütz, Elisa Jurianz, Hans-Günter Schaller, Stefan Reichert

**Affiliations:** Department of Operative Dentistry and Periodontology, Martin Luther University Halle-Wittenberg, Germany

## Abstract

**Background:**

Several studies suggest that there is a biologically plausible connection between rheumatoid arthritis (RA) and periodontal diseases (PD). Both disorders are characterized as multifactorial diseases potentially sharing common risk factors. Based on the inflammatory nature of RA and PD, the impact of genetic variations of genes of the immune system on both diseases was studied in this study.

**Materials and Methods:**

We conducted a case-control study (*n* = 201) comparing 101 RA patients suffering from periodontal disease of different severities (no/mild PD vs. severe PD) with 100 systemically healthy controls without RA and severe PD. The genotype, allele, and haplotype distributions of 22 SNPs of 13 pro- and anti-inflammatory cytokines were assessed applying sequence-specific PCR.

**Results:**

Evaluating the impact of cytokine SNPs in RA, we identified the G allele of rs1801275 in IL4R*α* (*p* = 0.043) and the G allele of rs361525 in TNF*α* (*p* = 0.005) as disease-associated risk factors in bivariate analyses. In multivariate analyses, these significant associations could not be proven. The A allele of rs2430561 in IFN*γ* was indicative for severe periodontitis among the patients with rheumatoid arthritis (*p* = 0.039). Investigating the impact of rs2430561 in IFN*γ* on comorbidity using binary logistic regression analyses, the A allele was confirmed as an independent risk factor for severe periodontal disease and RA (*p* = 0.024).

**Conclusions:**

These results emphasize the association of genetic variations in proinflammatory cytokines (TNF*α* and IFN*γ*) and cytokine receptor (IL4R*α*) and RA and periodontal diseases. In multivariate analyses, the A allele of IFN*γ* was proven to be a significant marker of RA and PD comorbidities. The study broadens the knowledge about disease-specific differences in genetic composition and provides an improved understanding of a possible association of both diseases.

## 1. Background

A relationship between periodontal disease (PD) and rheumatoid arthritis (RA) has been emphasized in several clinical studies [1–4]. Both diseases are described as chronic destructive inflammatory diseases sharing remarkable pathological and clinical similarities at cellular and molecular levels [5–7]. Patients suffering from rheumatoid arthritis are more likely to exhibit severe periodontitis or missing teeth than healthy controls [8–10]. On the other hand, patients with periodontal disease were shown to be more susceptible to RA compared with healthy persons [11]. There is a dose-dependent association pattern between severity of periodontitis and RA disease activity [3]. Moreover, the nonsurgical treatment of periodontal disease was shown to have a positive effect on rheumatic complaints [12, 13], and vice versa, the therapy of RA was proven to have a beneficial impact on periodontitis [14]. However, the possible underlying link between both diseases is not completely understood.

An important early clinical sign specific for RA is the occurrence of anti-citrullinated protein antibodies (ACPAs) [15]. It was demonstrated that the periodontopathogen *Porphyromonas gingivalis* (*P.g.*) has the unique capacity of expression of peptidylarginine deiminases, responsible for protein citrullination [16]. This fact corroborated the assumption of the involvement of periodontal infection in the aetiology of RA [17]. Genetic constellation (HLA-DRB^∗^04) was supposed to be associated with a higher odds ratio for the occurrence for borderline significance for ACPA (anti-CCP) [S. Reichert, personal communication]. However, no conclusive scientific evidence regarding the role of periodontal pathogens in RA was provided so far. Furthermore, it is recognised that increased inflammatory burden accompanied by PD and RA can mutually influence each other and affect further inflammatory diseases [1]. Of particular importance in this context are common features of regulation and dysregulation of inflammatory response [9, 18]. Periodontal disease and rheumatoid arthritis are characterized by excessive chronic inflammatory reactions leading to infiltration of T and B lymphocytes, neutrophils, and monocytes [19]. Both diseases are characterised by an imbalance between proinflammatory and anti-inflammatory cytokines [7, 20–22]. This complex interplay is a determining fact for PD and RA, respectively, and leads to the maintenance of inflammation and induction of bone resorption, joint destruction, and erosion [7, 23].

In recent years, a lot of clinical association studies were conducted in order to confirm the impact of genetic variants on RA [24, 25] and PD [26, 27]. Genetic studies reveal that both diseases are characterized by shared genetic risk factors such as a MHC class II HLA-DRB1 allele [28] or cytokine SNPs, including the KCNQ1 gene [29].

These considerations lead to the hypothesis of a shared genetic profile associated with a higher susceptibility to RA and periodontal disease. In order to support this hypothesis, we evaluated a panel of pro- and anti-inflammatory genes (IL1*α*, IL1*β*, IL1R, IL2, IL4, IL1RA, IL-4R*α*, IL6, IL10, IL12, IFN*γ*, TGF*β*, and TNF*α*) possibly involved in the aetiology of both inflammatory diseases. We assessed allele, genotype, and haplotype frequencies of these genes in RA patients suffering from PD of different severities and systemically healthy controls without RA and severe PD.

## 2. Materials and Methods

### 2.1. Study Population

In the case-control study, 201 unrelated subjects of Caucasian origin were included. In general, patients and controls were only included if they had a minimum age of 18 years (patients) or 30 years (controls), were not pregnant, and had not taken antibiotics in the past 3 months or undergone subgingival scaling and root planning procedures 6 months prior to the examination. The patients and controls had no known medical or general health conditions that might profoundly contribute to development of periodontitis (except RA in RA patients). In accordance with this, subjects were not included if they were suffering from diabetes mellitus type I or II, Morbus Crohn, coronary heart disease, lupus erythematosus, Behçet disease, or oral pemphigus or pemphigoid or if they developed gingival overgrowth due to specific drugs such as antiepileptics, calcium-channel blockers, or cyclosporine. The controls were excluded if they took anti-inflammatory drugs regularly. The medication of RA patients with nonsteroidal or anti-inflammatory drugs was recorded. During the course of anamnesis age, current or past diseases as well as medication and smoking status were assessed. The medication included nonsteroidal anti-inflammatory drugs (NSAIDs: 50%), disease-modifying antirheumatic drugs (DMARDs: 70%), and biologicals (42%). The periodontal examination comprised the assessment of approximal plaque index (API) [30], the percentage of sites with bleeding upon probing (BOP), pocket depth (PD: distance between the gingival margin and apical stop of the pocket), and the number of missing teeth. To determine the mean clinical attachment loss (CAL: distance between the cement-enamel junction and apical stop of the probe) in cases and controls, six sites around each tooth were measured, and the maximum values were recorded.

The control group is comprised of 100 subjects not suffering from rheumatoid arthritis. This group was recruited consecutively at the Department of Operative Dentistry and Periodontology of the Martin Luther University Halle-Wittenberg from 2005 until 2009. The periodontal examination was carried out by an experienced periodontist using a non-pressure-sensitive periodontal probe (PCPUNC156, Hu-Friedy, Rotterdam, Netherlands). All controls exhibit no or mild periodontitis according to the consensus report for “definition of a periodontitis case and disease progression in risk factor research” [31]. A “mild periodontitis” was defined as the presence of clinical attachment loss of ≥3 mm in ≥2 nonadjacent teeth. Controls with vestibular values of clinical attachment loss > 3.5 mm caused by traumatic tooth brushing or orthodontic therapy, CAL according overhanging subgingival restorations, or primary endodontic lesions were not considered as cases of periodontitis. Furthermore, pseudo pockets on the last molars with a depth of >3.5 mm were not considered as a periodontitis case [32].

The patient group includes 101 subjects with diagnosed rheumatoid arthritis according to current criteria for classifying rheumatoid arthritis [33]. RA was diagnosed and treated at the Clinic of Internal Medicine II, Department of Rheumatology, Martin Luther University Halle-Wittenberg (Prof. G. Keyßer and Dr. C. Schäfer), at the Department of Rheumatology “Rheumahaus Potsdam” (Dr. M. Bohl-Bühler and Dr. S. Reckert) and at three private practices in Magdeburg and Halle (Saale). From 2012 until 2016, the RA patients were included consecutively without consideration of periodontal status. For periodontal assessment, pressure-sensitive periodontal probes (TPS-probe Vivicare, Vivadent, Schaan, Liechtenstein, or DB764R Aesculap AG & Co. KG, Tuttlingen, Germany) were used. The dental examiners were instructed and trained in the implementation of both periodontal probes. The examiners were educated on a phantom model A-PB (frasaco GmbH, Tettnang, Germany) and under clinical conditions. According to the consensus report, a severe periodontitis case was defined as proximal attachment loss of ≥5 mm in >30% of the teeth [31]. Mild periodontitis was defined as mentioned above.

### 2.2. Genetic Investigations

For genetic investigations, fresh venous blood was obtained from the subjects in ethylenediaminetetraacetic acid- (EDTA-) treated tubes. Preparation of genomic DNA was carried out using a QIAamp blood extraction kit (Qiagen, Hilden, Germany) in accordance with the manufacturer's manual.

The analysis of SNPs was carried out using the Cytokine Genotyping array CTS-PCR-SSP Tray Kit (Collaborative Transplant Study, Department of Transplantation Immunology of the University Clinic of Heidelberg, Heidelberg, Germany). PCRs were performed with sequence-specific primers for the detection of genotypes and haplotypes. A fragment of 440 bp of the human C-reactive protein and 90 bp fragment of the human *β*-globin gene was coamplified as positive controls, respectively.

For every PCR, 10 ml of a Mastermix containing 1UTaq-Polymerase (Thermo Scientific, Waltham, USA), 100 ng genomic DNA, and PCR reaction buffer were added. PCR was performed in an Eppendorf Mastercycler Gradient (Eppendorf, Wesseling-Berzdorf, Germany) (2 min. 94°C; 10 cycles: 15 s 94°C, 1 min. 64°C, 30 s 72°C; 20 cycles: 15 s 94°C, 50 s 61°C, 30 s 72°C). After agarose gel electrophoresis, the resulting pattern was evaluated visually. In Table 1, the detected SNPs are displayed.

### 2.3. Molecular Biological Assessment of *Porphyromonas gingivalis*

Subgingival plaque samples were taken before subgingival scaling was carried out. The microbial samples were collected from the deepest pocket of each quadrant (insertion of a sterile paper point for 20 s) and pooled in one tube. Bacterial DNA was isolated applying the QIAamp® DNA Mini Kit (Qiagen, Hilden, Germany) according to the manufacturer's manual. The subgingival occurrence of *P.g.* was detected using the micro-Ident® test of HAIN-Diagnostik (Nehren, Germany) according to the manufacturer's protocol. The method was described in detail in [34].

### 2.4. Statistical Analyses

Statistical analyses were carried out using commercially available software (SPSS v.25.0 package, IBM, Chicago, IL). Values of *p* ≤ 0.05 were considered significant. Continuous data were assessed for normal distribution using the Kolmogorov-Smirnov test. These data were reported as means ± standard deviation (normal distributed values) or median, 25th/75th interquartiles (values not normally distributed). For the statistical evaluation of continuous variables, Student's *t*-test (normal distributed values), Mann–Whitney *U* test, or Kruskal-Wallis test (values not normally distributed) was used. Categorical variables were plotted in contingency tables and evaluated using the chi-square test and Yates continuity correction. If the expected cell frequency was <5, Fisher's exact test was applied.

Binary logistic regression analysis was used for investigating the impact of polymorphic variants on the occurrence of PD or RA considering established confounders.

## 3. Results

### 3.1. Clinical Evaluation

We performed a case-control study in order to evaluate the impact of genetic variants in selected pro- and anti-inflammatory genes (Table 1) in association with RA. We involved a control group of systemically healthy controls without RA and severe PD (*n* = 100) and a group of patients with RA (*n* = 101) suffering from periodontitis of different severities (severe periodontitis: *n* = 25; no/mild periodontitis: *n* = 76) in our study. The demographical and periodontal characteristics are displayed in Table 2. In general, patients suffering from RA were significantly older, were more often of female gender, and were more often smokers than probands without RA. Corresponding to the inclusion criteria, RA patients exhibited the more severe dental parameters including probing depth and clinical attachment loss. Subdividing the group of RA patients according to their periodontal status, it was obvious that patients suffering from severe periodontitis were more often males. In terms of age and smoking status, no significant differences were shown, although RA patients with severe periodontitis were older (*p* = 0.129) and more frequently smokers (*p* = 0.102) than RA patients with no or mild periodontitis. As expected, all RA patients suffering from severe periodontitis showed the more severe periodontal characteristics.

### 3.2. Genetic Variants in Rheumatoid Arthritis

Furthermore, we evaluated the impact of genetic variants in selected pro- and anti-inflammatory genes in association with occurrence of RA. Significantly, more G allele carriers of rs1801275 in IL4R*α* and of rs361525 in TNF*α* were in the group of patients suffering from RA compared to the group of probands without RA (Table 3).

In binary logistic regression analyses, including age, gender, smoking status, and the occurrence of *P.g.* as confounding factors, the G allele of IL4R*α* (*p* = 248) and the G allele of TNF*α* SNPs (*p* = 0.422) could not be proven as independently associated with RA. Increasing age, female gender, smoking, and the occurrence of *P.g.*, however, could be shown as significant risk factors for RA in these complex risk models (Table 4).

All other investigated genotypes, alleles, and haplotypes of pro- and anti-inflammatory cytokines were not found to be in association with rheumatoid arthritis in our study.

### 3.3. Cytokine Allele, Genotype, and Haplotype Frequencies in Association with Periodontitis

In a second evaluation, we tested possible associations between genetic variants and the severity of periodontal disease in the group of RA patients. No significant associations could be proven for all allele, genotype, and haplotype distributions investigated, except for IFN*γ* SNP rs240561. Patients suffering from more severe periodontitis were more frequently A allele carriers of this genetic variant than patients with no or mild periodontitis (*p* = 0.039, Table 5). In a multivariate risk model, higher age, male gender, smoking, and the higher incidence of *P.g.* but not the A allele of IFN*γ* SNP rs240561 had predictive value for severe periodontal disease. However, comparing patients with comorbidity of RA and PD with probands without RA, the A allele was significantly associated with both diseases in bivariate (Table 5, *p* = 0.039) and multivariate analyses (Table 6, *p* = 0.024).

## 4. Discussion

Over the last years, the involvement of genetic variants of cytokines as potential markers for disease susceptibility, progression, therapeutic success, and prognosis of PD and RA received particular attention [35, 36]. Based on the multifactorial pathogenesis of both diseases, it can be expected that genetic variants have rather a modulating than a determinant influence in this context. However, epidemiological studies showed that the genetic contribution to rheumatoid arthritis and periodontitis is substantial and may account for 50% of the RA [37] and PD risk profile, respectively [38, 39].

Therefore, we investigated possible associations between a panel of genetic variants of pro- and anti-inflammatory cytokine genes, including allele, genotype, and haplotype distributions, and RA or PD in bivariate and multivariate models considering further established risk markers for both diseases.

### 4.1. Clinical Evaluation

In the present association study, we included a group of controls without RA and severe periodontitis and a group of RA patients suffering from periodontitis of different severities (no/mild or sever periodontitis). As shown in Table 2, RA patients were significantly older than controls (*p* < 0.001). It is well established, that the incidence of rheumatoid arthritis increases with age [19]. Especially, women in their fourth and fifth decades are more affected by RA [40]. Also in our study, women were shown to be more susceptible to RA than men (Table 2, *p* = 0.002). Another major risk factor of RA is cigarette smoking. Smoking increases the RA risk especially in patients with genetic predisposition [41]. This fact was confirmed by our study, since RA patients were significantly more often smokers than controls without RA (Table 2, *p* = 0.005). As it was demonstrated by different studies, patients suffering from rheumatoid arthritis exhibit the more severe periodontal symptoms including probing depth and clinical attachment loss [6, 8].

Within the RA group, the patients were subdivided according their periodontal status. Patients suffering from severe periodontitis were older (n.s.), more often smokers (n.s.), and males (*p* = 0.014) compared to patients without or with mild periodontitis (Table 2). Since age, smoking, and the male gender are major risk factors of severe periodontitis, these tendencies were in accordance with established risk profile of periodontal disease [42].

### 4.2. Genetic Variants in Rheumatoid Arthritis

A lot of genetic variants have been reported to be implicated in the pathogenesis of rheumatoid arthritis [43, 44]. In this study, the G allele of SNP rs1801275 in the IL4R*α* gene and the G allele of SNP rs361525 in the TNF*α* gene were shown to be associated with RA out of 22 polymorphic variants in 13 cytokine genes (Table 3). However, considering further risk markers of RA, these associations of SNPs in IL4R*α* as well as in the TNF*α* gene and RA did not remain significant. This might indicate that factors like increasing age, the female gender, smoking, and the occurrence of *P.g.* were more strongly associated with RA implying a minor role of these genetic variants in our study.

Studies investigating the impact of rs1801275 in the IL4R*α* gene in RA revealed contradictory results [45–49]. In accordance with our results, Moreno et al. could confirm the G allele of rs1801275 as a risk factor for rheumatoid arthritis in RF positive patients [45]. On the other hand, other studies showed an impact of AG and AA genotypes [48] and A allele [49], respectively, on the development of RA. Furthermore, in a meta-analysis, no association of this genetic variant was demonstrated for rheumatoid arthritis [46] and for patients with juvenile idiopathic arthritis [47]. Since the inclusion criteria varied considerably, no consistent risk pattern regarding rs1801275 could be generated. It could be conceivable that genetic markers of IL4R*α* could influence its gene expression. This could be of great importance since IL4R*α* mediates the intracellular signalling cascades elicited by the anti-inflammatory cytokine IL4, a major regulator of the TH1/TH2 balance. However, functional studies could not prove a genetic influence of this polymorphism on gene expression in patients suffering from allergic asthma [50] or systemic sclerosis [51]. Further studies are needed in order to investigate the potential functional role of this polymorphism in rheumatoid arthritis.

Besides rs1801275 in the IL4R*α* gene, a genetic variant in the TNF*α* (G allele of rs361525) gene was shown to be associated with RA in our study. In a meta-analysis by Lee and Bae, the impact of A allele of rs361525 on RA was demonstrated evaluating 10 case-control studies including patients of different ethnicities [52]. However, taking only 6 studies conducted in Europe into consideration, the G allele was more frequent among RA patients (*p* = 0.047) [43]. These results obviously imply that the disease-related genetic characteristic of rs361525 is dependent on ethnicity. A possible genetic influence of rs361525 on TNF*α* expression was studied intensively leading to controversial outcomes [53–55]. However, in patients with rheumatoid arthritis [56] or osteoarthritis [57], the GG genotype and G allele of rs361525 were associated with increased TNF*α* expression, respectively. Therefore, in our RA group, the higher frequency of G allele carriers of rs361525 could be accompanied with higher TNF*α* expression indicative for RA [58]. Of particular importance for the RA therapy is the treatment with TNF*α* antagonists [59]. Indeed, it could be demonstrated that genetic variants in TNF*α*, including rs361525, were associated with responsiveness to TNF*α* treatment [60, 61].

Our results support the thesis of an association of genetic variants in cytokine genes (IL4R*α* and TNF*α*) with RA. However, in multivariate analysis, a corresponding genetic influence could not be proven (Table 4). This might imply that other risk factors are stronger disease-determining markers and that these genetic variants play a minor role in the aetiology of RA. And indeed, higher age, the female gender, smoking, and the subgingival occurrence of *Porphyromonas gingivalis* were significant predictors in binary logistic regression analysis. These results are in accordance with the established risk model of RA [19, 40, 41].

### 4.3. Cytokine Allele, Genotype, and Haplotype Frequencies in Association with Periodontitis within the Test Group

A lot of case-control studies, meta-analyses, and GWAS were conducted in order to evaluate the impact of genetic variants on the aetiology of aggressive and chronic periodontites with variable results [62–64].

A shared genetic background was assumed to be the basis among others for the biological plausible link between periodontitis and further inflammatory diseases, including RA. Therefore, studies were performed in order to identify common genetic risk factors for both diseases [14, 29, 65]. Applying the candidate gene approach, SNPs in IL1*β* (rs1143634) [13] and KCNQ1 (rs2237892) [29] were shown to be associated with comorbidity of rheumatoid arthritis and periodontal disease.

Also in this study, possible associations of genetic characteristics in cytokine genes and the severity of periodontal disease in RA patients were assessed. Out of the panel of 22 SNPs in 13 cytokine genes, we identified the A allele of SNP rs240561 in IFN*γ* as a risk indicator for severe periodontitis in RA patients (Table 5). Furthermore, evaluating patients suffering from RA and severe PD, the A allele was a significant predictor for comorbidity also considering further confounders (Table 6).

The scientific knowledge about possible association of rs2430561 to PD [66, 67] and/or RA [68–70] is highly inconsistent. Regarding possible association of this SNP and the susceptibility to PD, a meta-analysis was performed [71]. However, this study failed to prove a genetic association. The IFN*γ* SNP rs2430561 was demonstrated to be in complete linkage disequilibrium with a polymorphic microsatellite located in the first intron of the IFN*γ* gene with susceptibility to RA [68; 72]. In contrast, other studies failed to confirm this genetic association to RA susceptibility or severity [69, 70].

In the electrophoretic mobility shift assay, this SNP was demonstrated to be located in a putative nuclear transcription factor NF-*κ*B binding site influencing IFN*γ* expression [72]. Therefore, studies were conducted in order to assess the influence of this SNP on IFN*γ* expression resulting in different outcomes [72–75]. However, the inclusion and exclusion criteria of patients as well as the chosen methodological design varied widely. Several studies showed a lower expression associated with the A allele of IFN*γ* SNP rs2430561 [745]. In contrast, Prabhu Anand et al. could prove a higher IFN*γ* expression in peripheral blood mononuclear cells accompanied with AA genotype in healthy subjects [74]. Clinical studies investigating the impact of the IFN*γ* level on periodontitis showed an increased expression in saliva [76] and gingival biopsies in chronic periodontitis as well as in gingival crevicular fluid [77] in periodontal active sites [78]. Also, patients with rheumatoid arthritis were shown to exhibit higher levels of IFN*γ* in mononuclear cells and tissues from affected organs [79]. IFN*γ* and its associated signalling pathways were demonstrated to promote the breakdown of soft and hard tissues of the periodontium and induce bone loss [80, 81]. IFN*γ* is involved in the adaptive immune response due to the activation of macrophages and differentiation of T helper cells [82] including the induction of Th1 cytokines via the JAK/STAT signalling pathway [83]. These IFN*γ*-associated pathways are important characteristics in the aetiology of inflammatory diseases including PD [84] and RA [79], respectively. And indeed, the T cell-mediated increased IFN*γ* expression induced by periodontal infections with *P.g.* or *Aggregatibacter actinomycetemcomitans* was shown to be promoting rheumatoid arthritis [74]. Therefore, IFN*γ* and its functional important genetic variant rs2430561 could provide a biological plausible link between both inflammatory diseases.

### 4.4. Study Limitations

The present study was performed as a case-control study. It was conducted to establish assumptions of possible associations between genetic variants and periodontitis and rheumatoid arthritis, respectively. However, considering the study design, the verification of these assumptions is not realizable.

Due to one of the strengths of the present study, the homogeneous ethnicity, and due to the strict inclusion and exclusion criteria of the study participants, the sample size was relatively small. This may result in potential bias because of increasing the likelihood of a type II error skewing the outcomes. Therefore, the present investigation could only be considered as a pilot case-control study, and the drawn conclusions should be confirmed and extended in a large-scale cohort. Furthermore, the persons involved in the study are not necessarily representatives of the population as a whole.

In our study, 22 SNPs were tested. In chi^2^ tests, significant associations between genetic variants and PD/RA were assessed (Tables 3 and 5). However, if multiple hypotheses are evaluated, the likelihood of incorrectly rejecting the null hypothesis increases, which potentially leads to type I errors. Therefore, a statistical correction for multiple testing should be applied. After Bonferroni correction, the results of Tables 3 and 5 did not remain significant. Therefore, the drawn conclusion should be interpreted with caution.

Because of the integration of subjects from two settings, different methods of the determination of BOP, PD, and CAL were applied and were subject to possible biases. For RA patients, two different pressure-sensitive periodontal probes were used. The overall reproducibility of both probes has been already confirmed in previous studies [85, 86].

Control subjects were dental assessed using a non-pressure-sensitive periodontal probe. It has already been highlighted in earlier studies that both, non-pressure-sensitive periodontal probes and pressure-sensitive probes, are reliable tools for reproducible pocket depth measurements receiving a comparable error rate [87–89].

Finally, the data presented can be considered applicable for Caucasian individuals of central Germany only and must therefore be interpreted with caution. Extrapolation to the general population is not rationally supported.

## 5. Conclusions

Our results strengthen the thesis that SNP rs2430561 of the proinflammatory cytokine IFN*γ* may constitute a shared genetic risk factor for PD and RA. This might provide new arguments for the hypothesis of shared inflammatory processes underlying PD and RA. Further studies have to be conducted in order to replicate these findings in larger cohorts.

## Figures and Tables

**Table 1:** Genetic specificities of each gene investigated using the Cytokine CTS-PCR-SSP Tray Kit.

Gene	dbSNP-ID	Genotype/haplotype
IL1*α*	rs18000587	T/C
IL1*β*	rs16944	C/T
	rs1143634	T/C
IL1R1	rs2234650	C/T
IL1RN	rs31592	C/T
IL4R*α*	rs1801275	G/A
IL12B	rs3212227	C/A
IFN*γ*	rs2430561	A/T
TGF*β*1	rs1800470/rs1800471	CG, CC, TG, TC
TNF*α*	rs1800629/rs361525	GG, AG, GA, AA
IL2	rs2069762/rs2069763	TG, GG, GT, TT
IL4	rs2243248/rs2243250/rs2070874	TTT, TTC, TCT, TCC, GTT, GTC, GCT, GCC
IL6	rs1800795/rs1800797	GG, CG, GA, CA
IL10	rs1800896/rs1800871/rs1800872	GCC, GCA, GAC, ACC, ATC, ATA, ACA, ATA

**Table 2:** Demographical characteristics and periodontal conditions in relation to rheumatoid arthritis (RA) and periodontal disease (PD).

Variable	Probands without RA (*n* = 100)	RA patients			*p* value	
		All (*n* = 101)	No/mild PD (*n* = 76)	Severe PD^∗^ (*n* = 25)		
	I	II	III	IV	I vs. II	III vs. IV
*Demographical and anamnestic parameters*						
Age (years) (mean + SD)	45.8 ± 11.1	54.8 ± 13.1	53.6 ± 13.8	58.2 ± 10.3	**<0.001**	0.129
Female gender (%)	50	71.3	77.6	52	**0.002**	**0.014**
Current smoking (%)	20	24.8	21.1	36	**0.005**	0.102
*Periodontal conditions (median (25th-75th IQR))*						
Plaque index (%)	38 (28.7-59.3)	38.1 (16.1-68)	27.8 (9.5-57.8)	66 (41.5-83.2)	0.372	**<0.001**
Bleeding on probing/tooth (%)	42.4 (23.6-62.9)	38.5 (19.1-68.8)	32.7 (17.8-62.5)	60 (35.6-89.9)	0.934	**0.001**
Bleeding on probing/tooth surface (%)	8.7 (4.8-19.8)	9.5 (3.9-22.7)	8.6 (3.5-16.8)	19 (8.9-39.9)	0.664	**0.001**
Probing depth (mm)	2.5 (2.3-2.8)	4 (3-5.5)	3.5 (2.9-5.5)	5.5 (4.3-7.5)	**<0.001**	**<0.001**
Clinical attachment loss (mm)	2.8 (2.6-3.2)	4.1 (3.2-5.9)	3.5 (3-5)	5.9 (4.7-8.8)	**<0.001**	**<0.001**
Missing teeth (except 8th)	2 (0-3.75)	5 (2-10)	4 (1-9.75)	9 (5.5-15.5)	**<0.001**	**0.001**

^∗^Proximal attachment loss of ≥5 mm in ≥30% of teeth present. Statistical comparisons were made by the chi-square test including Yates correction for categorical variables. Continuous variables were analyzed by the Mann–Whitney *U* test and presented as median (25th/75th interquartiles (IQR); values not normally distributed) or Student's *t*-test and mean (standard deviation (SD); normal distribution).

**Table 3:** Genotype and allele distributions of SNPs in IL4R*α* (rs1801275) and TNF*α* (rs361525) among RA patients and controls.

	Probands without RA and severe PD	RA patients with varying degrees of severity of PD	*p* value
	*n* = 100	*n* = 101	
IL4R*α* rs1801275			
AA (%)	72	60	
AG (%)	25	33	
GG (%)	3	7	0.150

A (%)	84.5	76.5	
G (%)	15.5	23.5	**0.043**

TNF*α* rs361525			
AA (%)	1	1	
AG (%)	15	6.9	
GG (%)	84	92.1	0.186

A (%)	10	1	
G (%)	90	99	**0.005**

Statistical comparisons were made by the chi-square test including Yates correction. RA patients: patients with rheumatoid arthritis.

**Table 4:** Binary logistic regression analyses investigating the impact of G allele of rs1801275 (IL4R*α*) and G allele of rs361525 (TNF*α*) on the incidence of rheumatoid arthritis.

Variables	Regression coefficient	Odds ratio	95% confidence interval		*p* value
			Lower	Upper	
*IL4Rα (rs1801275)*					
Age	0.056	1.06	1.04	1.08	<0.001
Female gender	0.897	2.45	1.55	3.87	<0.001
Current smoker	0.841	2.32	1.48	3.64	<0.001
*P.g.* positive	0.655	1.93	1.18	3.13	0.008
G allele	0.336	1.40	0.79	2.47	0.248
*TNFα (rs361525)*					
Age	0.056	1.06	1.04	1.08	<0.001
Female gender	0.887	2.43	1.54	3.84	<0.001
Current smoker	0.863	2.37	1.51	3.71	<0.001
*P.g.* positive	0.676	1.97	1.21	3.19	0.006
G allele	0.370	1.45	0.59	3.57	0.422

Age, gender, smoking status, and the occurrence of *Porphyromonas gingivalis* (*P.g.*) were considered as confounding factors.

**Table 5:** Genotype and allele distributions of IFN*γ* SNP rs240561 in relation to severity of PD.

	Probands without RA and severe PD (*n* = 100)	RA and no/mild PD (*n* = 76)	RA and severe PD (*n* = 25)	*p* value		
	I	II	III	II vs. III	I vs. II	I vs. III
AA (%)	28.3	26.3	48			
AT (%)	44.4	50	40			
TT (%)	27.3	23.7	12	0.151	0.756	0.110
A (%)	50.5	51.3	68			
T (%)	49.5	48.7	32	**0.039**	0.966	**0.039**

Statistical comparisons were made by the chi-square test including Yates correction. PD: periodontal disease.

**Table:** (a) RA patients with severe PD vs. RA patients who do not have severe PD

Variables	Regression coefficient	*p* value	Odds ratio	95% confidence interval	
				Lower	Upper
Age	0.031	0.046	1.03	1.01	1.06
Male gender	1.149	0.002	3.15	1.53	6.49
Current smoker	0.893	0.024	2.44	1.13	5.3
*P.g.* positive	0.944	0.009	2.57	1.26	5.24
A allele	0.684	0.068	1.98	0.95	4.13

**Table:** (b) Patients with comorbidity of RA and severe PD vs. controls without both, RA and severe PD

Variables	Regression coefficient	*p* value	Odds ratio	95% confidence interval	
				Lower	Upper
Age	0.131	<0.001	1.12	1.08	1.17
Male gender	0.123	0.767	1.13	0.50	2.55
Current smoker	1.87	<0.001	6.49	2.69	15.6
*P.g.* positive	1.54	<0.001	4.67	2.07	10.5
A allele	0.977	**0.024**	2.66	1.14	6.20

Age, gender, smoking status, and the occurrence of *Porphyromonas gingivalis* (*P.g.*) were considered as confounding factors.

## Data Availability

The datasets used and/or analyzed during the current study are available from the corresponding author on reasonable request.

## Abbreviations

API: Approximal plaque index
BOP: Bleeding upon probing
CAL: Clinical attachment loss
DMARD: Disease-modifying antirheumatic drug
GWAS: Genome-wide association study
IFN*γ*: Interferon gamma
IL4R*α*: Interleukin 4 receptor alpha
n.s.: Not significant
NSAID: Nonsteroidal anti-inflammatory drug
PD: Periodontal disease
*P.g.*: *Porphyromonas gingivalis*
RA: Rheumatoid arthritis
SNP: Single nuclear polymorphism
TNF*α*: Tumor necrosis factor alpha
vs.: Versus.
